# The Role of the Sentence Constraint in New Word Acquisition While Reading in Adolescents: The ERP N400 and P600 and Reading-Related Skills

**DOI:** 10.3390/brainsci15060607

**Published:** 2025-06-04

**Authors:** Marina Norkina, Anna Rebreikina, Maksim Markevich, Elena L. Grigorenko

**Affiliations:** 1 Center for Cognitive Sciences, Sirius University of Science and Technology, 354340 Sirius, Russia; anna.rebreikina@gmail.com (A.R.); markevich.mo@talantiuspeh.ru (M.M.); 2 Institute of Higher Nervous Activity and Neurophysiology, 5A Butlerova Str., 117485 Moscow, Russia; 3 Department of Psychology, University of Houston, 126 Heyne Bldg, Houston, TX 77204, USA

**Keywords:** new word acquisition and word recognition, context constraint, adolescents, ERP, N400, P600, LPC

## Abstract

Background/Objectives. Vocabulary acquisition is a lifelong process, with the most rapid growth occurring from early childhood to school age. Different contextual factors influence how new vocabulary is acquired across various age groups during reading. Methods. We studied the process of new word acquisition in different constraining contexts in adolescents aged 11–17 years old and how individual differences in reading comprehension, vocabulary, and verbal working memory affect word acquisition. In the learning stage, the new words were presented in sentences with low and high contextual constraints, and word acquisition was assessed in a word recognition test where behavioral measures and the N400 and P600 components of the event-related potentials (ERPs) were examined. Results. Our study reveals that while the accuracy of word recognition was at a chance level, adolescents had faster responses to words learned in high-constraining contexts compared to words from low-constraining contexts. Neural responses were influenced by context, with explicit recollection processes reflected in the P600 being modulated by the type of sentence constraint, while implicit familiarity related to the N400 did not show this effect. Higher reading comprehension, vocabulary, and verbal working memory scores improved accuracy, while reaction times were improved by just vocabulary. Additionally, reading comprehension and vocabulary impacted the implicit N400 old/new effect, and reading comprehension correlated with explicit recognition processes (P600 old/new effect). Conclusions. Therefore, the present study showed that the type of constraint of new word learning and individual skills affected the word acquisition process in adolescents.

## 1. Introduction

Learning new words is a lifelong process that occurs at different stages of human development, from childhood to adulthood. It is most rapid from early childhood to school age [1]. The process of vocabulary acquisition is very fast, and children develop skills from using their first words to constructing sentences. Vocabulary continues to grow in school as new words are learned from context during learning, especially reading [2].

There are different factors that influence the process of new word acquisition at different stages of life. In adolescence, the main source of learning new words is the text context. As early as primary school, children begin to learn from the context, mostly while reading or listening, usually without getting explicit definitions of the new words [3]. This type of vocabulary instruction requires the determination of the meaning of the new word based on the surrounding linguistic context [4,5].

Therefore, the context and its characteristics are important factors in learning research, especially in the acquisition of new words. The comprehension of the words during reading may depend on the sentence constraint. If the context leads to the particular word completion at the end of the phrase or sentence, this type of constraint is called ‘high-constraining’. For example, “Grandmother put on a warm…”; the sentence may be continued with a particular item of clothing. In a low-constraining context, the candidate for the ending is not so specific [6]. For example, “A woman asked about a...”. It should also be noted that the context type and its constraint may have different terms across studies and various paradigms. In some works, terms such as ‘supportive’ and ‘nonsupportive’ are used rather than the level of constraint [7,8] or ‘high predictive’ versus ‘low predictive’ context [9]. A supportive context allows the reader to infer the meaning of the word, whereas a nonsupportive context does not lead to a specific meaning. Thus, despite the different terms, these listed definitions refer to the predictability and structure of sentences from which the meaning of a particular word could be extracted and understood.

The studies on the acquisition of new words in different contexts vary in terms of the tasks and investigated age range [10]. At the behavioral level, it has been shown that context can improve learning [11,12,13,14,15,16]. However, the results on the robust effect of learning from context while reading are quite controversial [2,8]. It is argued that a single exposure does not lead to the acquisition [17] but allows partial recognition and/or retrieval of meaning [8,18].

Word acquisition goes through several stages, from complete unfamiliarity to use in context. Behavioral methods capture this complex process of new word acquisition to some extent. Neuroimaging methods, with online registration of the process in milliseconds, shed additional light on this process in much more detail [19]. Word learning and its neural correlates have been studied at different stages of word acquisition (e.g., word recognition, meaning retrieval, and memory consolidation) [7,9,20]. An event-related potential (ERP) recording consists of several distinct components or brain waves corresponding to different stages of stimulus processing. Numerous studies have shown that the neural correlates, including N400 and P600, could be potential candidates for the neural markers of new word learning at different acquisition stages [7,8,21], e.g., N400 showing lexical-semantic and familiarity effects and P600 recollection [2,19]. The results of electroencephalogram (EEG) data on a neural basis have also shown significant differences in the brain’s response to newly learned words from different contexts. Most of the studies have employed various experiments with context manipulation and testing after the learning phase [7,9,18,22].

In general, the N400 has been usually associated with lexical-semantic information processing [23], but studies of word memorization have also linked this component to familiarity [19]. The N400 is less prominent for ‘old’ (memorized) words than for new words. With regard to contextual constraint, results in adults (M = 21; 7 years old) showed a reduction in the N400 component for words presented in supportive contexts compared to those presented in unsupportive contexts [7]. It is noted that age is an important factor influencing language learning processes and the development of predictive processing [18]. Children’s behavioral and neural performance during contextual word learning has been investigated but to a much lesser extent than adults [10]. One of the studies with children (M = 9; 4 years old) was conducted by Abel and colleagues [8] at the behavioral and neural levels. The results indicated that the participants were not consistently able to discriminate between familiar and new nonsense words in terms of their behavior, despite the supportive meaning or the meaningless context. However, the N400 ERP component analysis shows that nonsense words presented with meaning elicited a less negative N400 amplitude compared to those presented without meaning, while the unfamiliar nonsense words showed no significant differences. The study in children and early adolescents [9] showed that the effect of the novel word on the N400 amplitude was influenced by both the context constraint (high or low) in which the new words were presented and the age of the participants. Thus, the observed alteration in brain activity associated with the N400 component is proposed to serve as an indicator of the real-time processing and comprehension of the meaning of a novel word derived from contextual cues [7]. This phenomenon has been studied in various age groups, encompassing both children and adults, but remains understudied in adolescents.

Another ERP component, the late positive complex (LPC or P600), is characterized by a positive deflection that peaks around 600 ms after stimulus presentation. Stimuli perceived as old (i.e., encountered earlier in an experimental study phase) generally produce a more positive LPC compared to those perceived as new, indicating recollection [24,25]. Recollection involves retrieving contextual details from a past event, and it is linked to an awareness of that retrieval [2]. The P600 has also been investigated in contextual studies [22,26]. Balass and colleagues [26] investigated the effects of different word-learning contexts on word identification and meaning retrieval in college students using behavioral assessments and ERP recordings. Their findings indicated that high-skilled readers showed larger positive P600 amplitudes for learned words compared to less-skilled readers, who struggled to form quality representations and failed to recognize previously encountered words, potentially hindering their future learning.

In the studies of context constraint and the acquisition of new words, the most studied age is adult, the age when reading-related skills are already developed, and to a lesser extent, children when the skills are actively being formed; the least studied age is adolescence. However, adolescence is a transitional age when literacy skills are being formed and adulthood is approaching, such as reading, considering that adolescents in some studies exhibit difficulties in reading comprehension [27,28]. It is a crucial age for investigating performance in word acquisition from the context during reading at both behavioral and neural levels. Thus, it remains uncertain how likely adolescents are to use prediction to associate meanings with new word forms or how much linguistic experience they require to accomplish this [29,30]. The current study addresses these issues by examining both behavioral measures and event-related potentials’ recording in response to the acquisition of new words within sentence contexts. It also varies the level of contextual constraint (low- and high-constraining contexts) to elicit either partial or strong word knowledge.

Individual differences are the focus of the research, as it has been shown that readers who struggle with reading comprehension have difficulty understanding the meaning of words [2,8,26,31,32]. It was shown that a smaller vocabulary also leads to difficulty focusing on targets presented in sentences with low-constraining sentences compared to those with high-constraining sentences [33,34]. More broadly, challenges in word-learning tasks may be related to limitations in cognitive resources, such as working memory, which may hinder the ability to effectively infer new word meanings [35].

Therefore, in addition to using ERPs to reveal the effects of new word acquisition in different constraining contexts, we are also investigating the question of individual differences in comprehension skills toward new word acquisition. The results of the study will advance our understanding of literacy-related neurodevelopmental disorders, in particular, the relationship between cognitive processes—such as reading comprehension, verbal working memory, and vocabulary size—and new word acquisition.

Our study aims to address three primary research questions:

1. Does contextual constraint influence new word acquisition at the behavioral level in adolescents?
2. Do different sentence constraints modulate ERPs in adolescents during new word acquisition, and at which processing stages?
3. Are behavioral and neural results related to individual reading-related skills in adolescents?

### Hypothesis

We predict that exposure to new words in a high-constraining context would lead to better word recognition compared with a low-constraining context; moreover, this advantage will be reflected at both behavioral and neural levels. At the behavioral level, reaction time and accuracy will be faster and more accurate, respectively, for words learned in the high-constraining context. At the neural level, words acquired in a high-constraining context would elicit differences in ERP patterns compared to words learned in the high-constraining context: N400 and P600. We predict that the words learned in the high-constraining context will result in less negative N400 amplitudes and higher P600 components compared to words from low-constraining contexts and distractors (new words not presented in the learning phase of the experimental task). In terms of individual differences, we hypothesize that the participants with high scores on the verbal working memory and reading-related tests will show more pronounced behavioral and ERP constraint context effects.

## 2. Materials and Methods

The study protocol was approved by the Institutional Review Board of the Sirius University of Science and Technology, Sochi, the Russian Federation (protocol reference number from 31 August 2021). Prior to this study, informed consent was obtained from both the children and their legally authorized representatives. All aspects of the research conformed to the tenets of the Declaration of Helsinki. The current study was conducted as part of the larger study of “Literacy as a Foundation of the Knowledge Economy: A Neuroscientific Approach”, project agreement 075-10-2021-093, COG-RND-2138, which aims to elucidate the mechanisms of reading (dis)ability in adolescence. The results of a pilot experiment conducted in the studies have been reported [36,37,38].

### 2.1. Participants

A total of 58 participants aged 11–17 years (Mage = 15.34 years, SD = 1.37, 34 females) were recruited for this study. All the participants were enrolled in public schools in different regions of Russia. A total of 15 participants were recruited locally, either through schools or through social media and flyers. At the time of this study, 43 participants from different regions of Russia were involved in a range of educational programs—including “Biology”, “Informatics”, “Natural Science”, “Physics”, and “Chemistry”—at the Sirius Educational Center, where they were subsequently recruited for this study. Data from 3 participants were excluded due to an excessive number of EEG artifacts (>30% of all epochs). This left 55 participants for the final analysis, ranging in age from 11 to 17 years (Mage = 15.39 years, SD = 1.28, 31 females). All the participants were monolingual native Russian speakers with normal or corrected-to-normal vision; they had no history of mental illness, language impairment, drug abuse, or neurological trauma, and none of the participants reported reading difficulties. The demographic characteristics, reading habits, and language environment of the participants are presented in Table A1 and Table A2 in Appendix A.

Both the participants and their parents were informed of the nature and duration of the experiment, as well as their right to withdraw consent at any time. Parents provided informed consent by signing forms expressing their wish for their children to participate in this study. In appreciation of their participation, the children received bookstore gift vouchers and souvenirs, and volunteer honor hours were granted if requested.

### 2.2. Design

The participants completed several tasks, including behavioral and psychophysiological blocks. The experimental paradigm was presented in the psychophysiological block. The behavioral and psychophysiological blocks were randomized and, due to the length of the session and the availability of the participants, took place within 1 day to 1 week of each other.

#### 2.2.1. Individual Reading-Related Skills

Each participant’s reading comprehension, vocabulary (synonym task), and verbal working memory (wordspan and pseudowordspan task) were assessed using methods from the project “Literacy as a Foundation of the Knowledge Economy: A Neuroscientific Approach.” A summary of the descriptive statistics for these measures is provided in Appendix A Table A3.

Reading comprehension was assessed using a task adapted from the PISA 2018 framework [25], in which participants silently read three mixed texts (650–750 words each) containing both continuous (e.g., novels, newspaper reports, essays, reviews, letters, and short stories) and non-continuous (e.g., graphs, lists, tables, advertisements, schedules, diagrams, indices, and catalogs) texts, and answered 10 corresponding questions (closed-ended and open-ended) per text to assess information localization, content comprehension, and evaluation via a web platform on laptops or tablets.

In the auditory Wordspan and Pseudowordspan tasks, the participants were presented with lists of two- to eight-syllable nouns (or pseudowords) that were controlled for length and frequency, with the list length increasing stepwise after a two-item presentation. The participants listened to the lists of words and were asked to repeat them aloud. A key requirement was that they could not begin their response with the last word they had heard on the list. The total number of words correctly reproduced in each subtest was assessed.

In the synonym task, part of a new Russian literacy assessment [39], the participants were orally presented with 50 words (nouns, adjectives, and verbs) of different frequencies (high, medium, and low frequency) and asked to identify a synonym, with correct responses being summed to determine performance, with stimuli and responses validated by experts. The tasks used are also described in more detail in Berlin Khenis and colleagues [37].

#### 2.2.2. The Experimental Paradigm

The experiment consists of two phases: a learning phase—the presentation of a new word (pseudoword) in two conditions: constraint condition (sentences varying gradually from low-constraining to high-constraining context) and control condition (sentences in low-constraining context only) and a testing phase (immediately followed the learning phase)—‘the old/new” word recognition task, where the acquisition of new words was checked.

**Figure 1:**
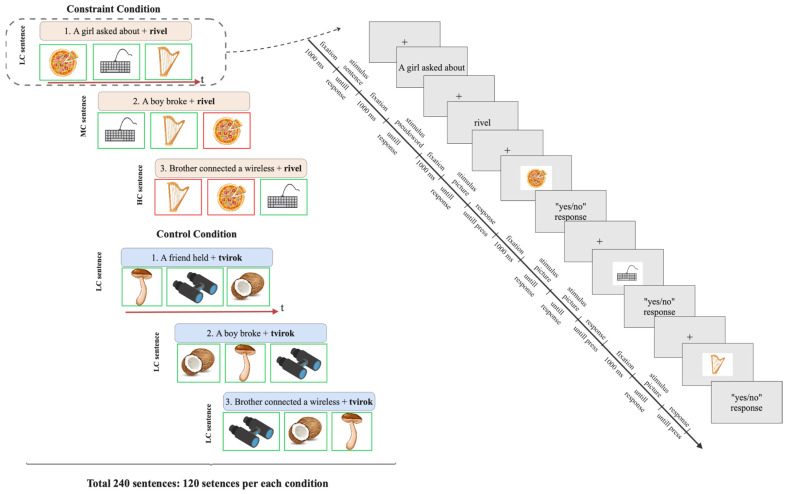
The block-scheme of the learning phase. The left figure illustrates the blocks for the constraint condition and control condition. Each block included 3 sentences with a new word (pseudoword) in the terminal position presented separately on the screen. After each sentence, three pictures were presented sequentially to each of which the participant had to give a response. The green frame on the scheme marks the pictures corresponding to the new word, and the red frame marks the pictures not related to the word. The right figure represents the time course of the stimuli presentation and responses in the one trial of the block.

In the learning phase, the stimuli consisted of 80 new words (pseudowords): 40 new words were presented in the constraint condition, and 40 new words were presented in the control condition. The experimental paradigm’s design is presented in Figure 1 (the learning phase). Each new word was presented in a sentence. All the sentences were in Russian and were followed by three pictures. The participant’s task was to decide, based on the sentence contexts, whether the following picture could represent the possible meaning of a presented new word (pseudoword). The stimuli were arranged in triplet sentences: in 40 blocks of trials for the constraint condition and 40 blocks for the control condition. In the constraint condition, the sentence constraint was manipulated; the trial block consisted of a low-constraining sentence, a moderately constraining sentence, and a high-constraining sentence (3 sentences * 3 constraint types; 120 sentences in total). Following the initial sentence (low-constraining sentence), three pictures were presented one at a time; the correct response for each of the presented pictures was “yes”, meaning the picture could correspond to the new word (pseudoword). However, in relation to the second sentence, two of the three pictures were relevant, while only one picture corresponded to the meaning conveyed in the third sentence. All sentences in the control condition set were low-constraining (3 sentences * 1 constraint type; overall 120 sentences). Pictures presented after sentences in the control condition were all relevant to the new words (pseudowords). Both sets contained a pseudoword as the target new word in the final sentence position. The sentences in each block of trials were semantically unrelated. The blocks of the constraint condition and of the control condition were presented in pseudorandom order.

**Figure 2:**
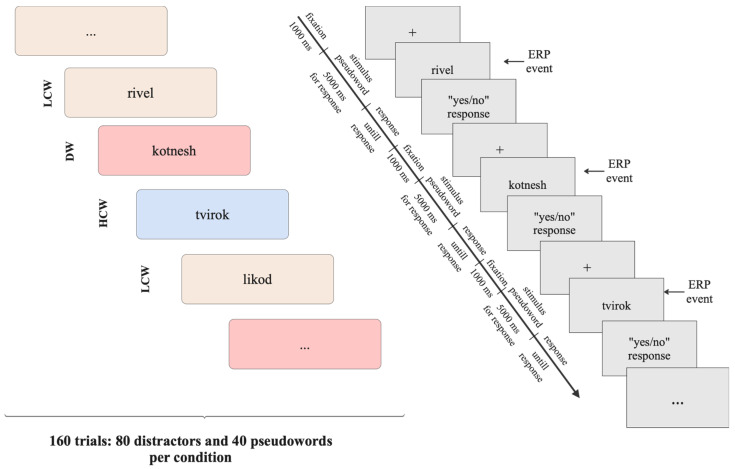
The block-scheme and timeline of the testing phase. The block-scheme (on the left) represents the design of the Word Recognition test in a total of 160 trials. There were three types of stimuli: distractors (DWs) (not presented in the learning phase pseudoword), 40 words from a highly-constraining context (HCW), and 40 words from a low-constraining context (LCW). The timeline (on the right) represents the time course of the word recognition task.

In the testing phase, 160 words were presented: 80 new words were distractors (DWs) (were not presented in the learning phase), and another 80 words were the new words presented in the learning phase (40 words from a highly-constraining context (HCW), i.e., from the constraint condition; and 40 words from a low-constraining context (LCW), i.e., from the control condition). After the first half of the task, there was a short, self-paced break to allow the participants to rest. The stimuli were randomized. The experimental paradigm’s design is presented in Figure 2 (the testing phase).

### 2.3. Stimuli

#### 2.3.1. The Learning Phase

Sentence constraint: Low- and moderately constraining sentences were simple, non-extended sentences with the SVO (subject–verb–object) structure. High-constraining sentences were simple non-extended sentences with the SVAO (subject–verb–adjective-object) structure. Adjectives in high-constraining sentences helped to narrow the meaning of a pseudoword. Examples of sentences in each condition are presented in Table A4 in Appendix A. A detailed description is provided by Semenova and colleagues [38].

Pseudowords stimuli: We used the online tool “Pseudo” [40] to create a set of 80 pseudowords. Pseudowords were either disyllabic or trisyllabic and consisted of 4 to 8 letters; they were categorized as neuter or feminine gender and equalized in the way that each gender was presented the same amount of times.

Pictures: All images were sourced from the Vecteezy marketplace and feature licensed vector graphics. The images were consistent in style and size, and they were arranged on a white background using the Pixlr editor [41]. The resolution of the images was 2160 × 1620 pixels.

#### 2.3.2. The Testing Phase

The distractor stimuli were created in accordance with the pseudowords from the learning phase. To create the distractor stimuli for the testing phase, we first selected 80 real Russian words that were frequent concrete nouns (disyllabic or trisyllabic), written in 4–8 letters. Then, we composed novel words by recombining the onsets and offsets of these real words to create novel, meaningless word forms.

All the stimuli are openly available via the Open Science Framework (OSF): https://osf.io/tdzgv/?view_only=65916d48c67e423e8c238e037bd63ccf, accessed on 1 June 2025.

### 2.4. Experimental Procedure

The participants were seated at a distance of approximately 75 cm from a computer screen in a sound- and light-insulated room. The experiment was run using the Psychopy 2021 software, version 3.2.1 [42], and presented on the Lenovo Legion screen (resolution: 1920 × 1080; diagonal: 17.3″). Task instructions were displayed on the screen and explained by the assessor.

The experiment was conducted in two parts, with a break in between, and lasted approximately 60 min in total (see Figure 1 and Figure 2 for an example of the timeline of the experiment for the learning phase and testing phase, respectively). In the learning phase, participants were instructed to read sentences that concluded with a target new word (a pseudoword). After each sentence, they were presented with three pictures in sequence and asked to indicate whether each picture corresponded to the meaning of the new word. To respond, they pressed either the green key or the red key on the keyboard (indicating “yes” or “no”). The results of the learning phase of the experiment were analyzed separately and are not presented in the current study.

In the testing phase, the word recognition test was presented. In this task, the participants were asked to answer whether the word (pseudoword) was presented in the sentences in the previous task by pressing either the green key (for “yes”) or the red key (for “no”) on the keyboard. The stimuli were presented in the center of the grey screen and displayed in black Open Sans font. Each word was presented until a response was made, with a time limit of 5 s for a response, after which the next word was presented. A fixation cross was displayed for 1000 ms before each stimulus. The order of presentation was randomized.

### 2.5. EEG Recording

The EEG signal was recorded with 128 Ag/AgCl active electrodes (ActiCAP, Brain Products GmbH, Gilching, Germany [43]) configured according to the international 10–5 system and a sampling rate of 500 Hz. The impedance was kept below 10 kOhm. Data were recorded with the ground at the central frontal electrode and the reference at FCz.

### 2.6. Data Pre-Processing and Analysis

#### EEG Pre-Processing

The EEG signals were analyzed using a Brain Vision Analyzer (BVA, version 2.2.1) [41]. EEG records were filtered with the Butterworth Infinite Impulse Response (IIR) Filter, a low cut-off—0.1, a high cut-off—70 Hz, and 50 Hz Notch filters, and an 8th filter order setting (corresponding to 48 dB/octave). The bad channels were interpolated with spherical spline-type interpolation prior to the oculographic correction and re-referencing. The criteria for interpolation were that the activity was higher than 1000 µV on more than one-third of the channel recording. Segments with bad recordings exceeding ±400 mV were removed from the analysis. Artefact Fast ICA was used to correct oculographic and other artefacts (such as pulse and regular local activity of unclear genesis) on the continuous data before segmentation. The artifactual components were identified with visual inspection based on topography and time course. Then, the data were re-referenced to an average reference. Data were segmented into segments of −200 to 1500 ms relative to each stimulus (pseudowords) in the testing phase. The baseline was corrected using the pre-stimulus time window (−200–0 ms), and the removal of artefact segments were performed (+/−110 mV). ERPs were averaged for each condition regardless of the correctness of the answer (due to the low correct response for each condition, the mean correct response was 56%, which resulted in a small amount of the segments left for analysis). The conditions were DW, HCW, and LCW. To average all the conditions on an equal number of the segments, the DW condition was averaged based on half of the stimuli (40 out of 80 trials). The mean number of averaged epochs was M (SD) = 34.28 (4.79) (see descriptive statistics of epochs for each condition in Appendix A Table A5).

### 2.7. ERP Calculation

**Figure A1:**
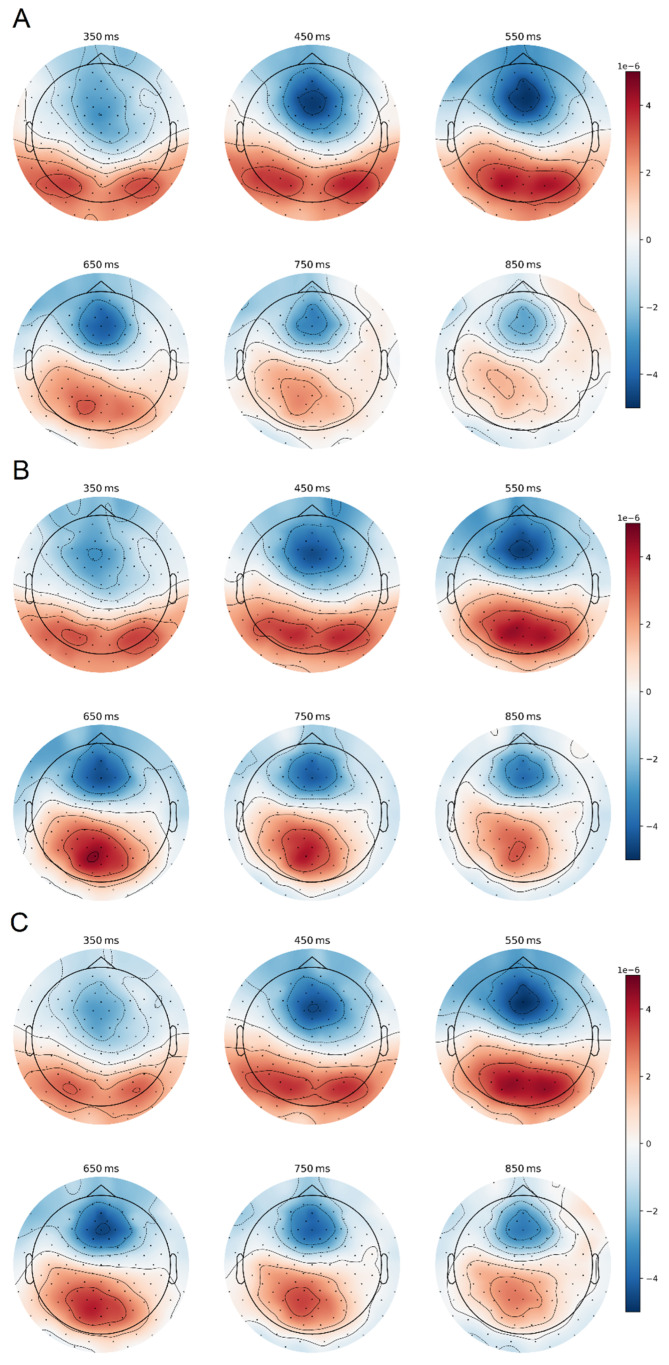
Topography of visual evoked potentials in response to distractor words (**A**), high-constraining words (**B**), and low-constraining words (**C**).

We operationalize N400 and P600 as the mean amplitudes between 350–450 ms and 600–800 ms averaged across the centro-frontal electrode sites (C1, C2, C3, C4, FC1, FC2, FC3, FC4, FCC1h, FCC2h, FCC3h, FCC4h, FCC5h, and FCC6h) and parieto-occipital electrode site (O1, O2, P1, P2, P3, P4, PO3, PO4, POO1, POO2, PPO1h, and PPO2h), respectively. For topographic maps of the components, see Appendix A Figure A1. The N400 and P600 time windows were chosen based on the maximal expected ERP response locations based on prior literature [44,45,46] and based on the visual inspection of ERPs and the global field power (GFP) across all scalp electrodes demonstrating a clear, sharp peak within that specific interval for N400 and a distinct interval for P600.

### 2.8. Analysis

Statistical analysis was performed using the “lmer4” package [47] in the R programming environment, version 4.3.1 [48]. Post-hoc tests were conducted using the “emmeans” package with Tukey’s adjustment method.

#### 2.8.1. Behavioral Data

##### Accuracy Response

A logistic mixed-effects model (GLMER) was utilized to analyze the accuracy of the experiment task. The logistic model was performed to investigate the effect of the condition (DW, HCW, and LCW) (fixed effect) on the likelihood of participants’ answer on the word recognition test (binary response: correct vs. incorrect response), while accounting for random effects due to variations among participants and trial of the word presentation (random intercepts) (Model 1: glmer(Response accuracy ~ Condition + (1|ID) + (1|TRIAL), data = data, family = binomial)).

To control for response bias (i.e., a tendency to respond “yes” or “no” regardless of stimulus), we employed signal detection theory [49] to calculate discrimination sensitivity (d’) and response bias (c) for each participant across all conditions. d’ was calculated as the difference between z-transformed hit and false alarm rates, while c was calculated as −0.5 × (z(hit rate) + z(false alarm rate)). Hit rate is the proportion of times a participant correctly identifies stimuli that were previously presented. False alarm rate indicates the proportion of times a participant incorrectly identifies distractor stimuli as previously presented. Correct rejections refer to the proportion of times a participant correctly identifies that a distractor stimulus was not previously presented. Misses represent the proportion of times a participant fails to recognize stimuli that were presented.

##### Reaction Times

All observations in reaction times (RTs) that fell outside of 2.5 standard deviations from the mean were considered outliers (3.37%). The logarithmic transformation was used to standardize the RTs. To investigate the factors influencing RTs, a linear mixed-effects model (LMER) was employed (Model 2: lmer(Reaction time ~ Condition*Response accuracy + (1|ID) + (1|TRIAL), data = data, REML = FALSE, control = lmerControl(optimizer = “bobyqa”)).

The selected approach allowed us to model both the fixed context constraint effect and individual-level variability. The condition was a factor variable with 3 levels (DW, HCW, and LCW), with the HCW condition serving as the reference level in both models (accuracy and RTs).

#### 2.8.2. Neurophysiological Data

We implemented linear mixed-effects models (LMER) to test if the different sentence constraints modulate ERPs in adolescents while new word recognition during the test phase. LMER models (Model 3 and Model 4) with N400 and P600 mean amplitudes between 350–450 and 600–800 ms, respectively, were used to test the hypothesis that different sentence constraints modulate the ERP responses to the words during the word recognition task. Random effects were included in the model: participants and electrodes. For fixed effects, conditions (DW, HCW, and LCW), brain hemispheres (right and left), and participants’ task accuracy mean scores were used. Due to the small amount of segments for each condition of just correct responses, ERPs for each condition were averaged across all responses. Therefore, the accuracy score was included in the models to account for the variability related to performance differences between participants on ERPs. The interactions between fixed effects were included in the models. The condition was a factor variable with 3 levels (DW, HCW, and LCW), with the HCW condition being the reference level in the models. For the factors of hemispheres, we used a sum-to-zero contrast matrix, so the grand mean across factors was included in the intercept. The accuracy variable was centered and scaled.

Model 3: lmer(N400 Mean amplitude ~ Condition*Hemisphere*Accuracy + (1|ID) + (1|ELECTRODE), data = data, REML = FALSE, control = lmerControl(optimizer = “bobyqa”)).

Model 4: lmer(P600 Mean amplitude ~ Condition*Hemisphere*Accuracy + (1|ID) + (1|ELECTRODE), data = data, REML = FALSE, control = lmerControl(optimizer = “bobyqa”)).

#### 2.8.3. Individual Differences and Correlations

To answer the question about the role of individual reading-related skills on behavioral and neural levels in adolescents, we analyzed the scores of the cognitive reading-related skills’ tasks: reading comprehension, vocabulary, and verbal working memory. To investigate the effects of the subtests performance (reading comprehension, vocabulary, and verbal working memory) and the results on the response accuracy and reaction times, a linear model was used. (Model 5: lm(Accuracy response/RTs ~ Subtest Performance + Age)). To explore the effects of the subtests performance (reading comprehension, vocabulary, and verbal working memory) and the results on the neural level, a linear mixed-effects model (LMER) was conducted. For dependent variables, the differences in the mean amplitudes of the N400 to HCW and to WD, as well as the differences in the amplitude of the P600 to HCW and to WD, were used. The differences in mean amplitudes of ERPs of the significant results for the main effects of the condition were tested only. (Model 6: lmer(Diff wave ~ Subtest Performance + Age + (1|ID) + (1|ELECTRODE), data = data, REML = FALSE, control = lmerControl(optimizer = “bobyqa”)). All behavioral variables, including age, were centered and scaled, calculating z-scores.

## 3. Results

### 3.1. Behavioral Data

To assess the behavioral results of the word recognition task, we analyzed the accuracy of the response as the number of correct answers for each condition. The reaction times to the response were also evaluated for each condition. Mean accuracy scores and reaction times on the task are shown in Table 1.

#### 3.1.1. Accuracy Results

The model results revealed a significant effect of the condition. Specifically, the participants gave significantly less correct responses to the HCW compared to LCW and DW (LCW: z = 2.00, *p* = 0.04; DW: z = 17.22, *p* < 0.000) (see Table 2). The results of post hoc tests are presented in Appendix A Table A6.

The analysis of discrimination sensitivity (d’) and response bias (c) revealed means and standard deviations of hits (M = 44.73 and SD = 10.42) and false alarms (M = 19.44 and SD = 11.29). The high combined rate of false alarms and misses indicates that some participants may have exhibited a response bias. Specifically, a mean c bias of 0.31 (SD = 0.36) suggests a tendency towards greater inclination to respond “no”, meaning that we cannot definitively rule out the possibility that some responses, particularly those related to words from the learning phase, were not solely based on accurate discrimination but also influenced by a participant’s predetermined response strategy. For complete descriptive statistics, refer to Table A7 in Appendix A.

#### 3.1.2. Reaction Times Results

The model results revealed a significant effect of the condition (see Table 3). Specifically, the participants gave significantly faster responses to the HCW compared to LCW (t = 2.50, *p* = 0.01) and faster compared to DW (t = 2.88, *p* = 0.00). The accuracy factor was also significant (t = −2.52, *p* = 0.01). The results of post hoc tests are presented in Appendix A Table A8 and Table A9 and reveal the interaction of accuracy and reaction time between the conditions. In the incorrect responses, there was no significant difference in reaction time between the conditions. In the correct responses, reaction time was faster for HCW (t = −2.58, *p* = 0.03) compared to DW (Table A8 and Table A9 in Appendix A). There were no significant differences for HCW compared to LCW (t = −1.50, *p* = 0.29) and LCW compared to DW (t = −0.80, *p* = 0.70).

### 3.2. Neurophysiological Data

#### 3.2.1. N400 Mean Amplitude

**Figure 3:**
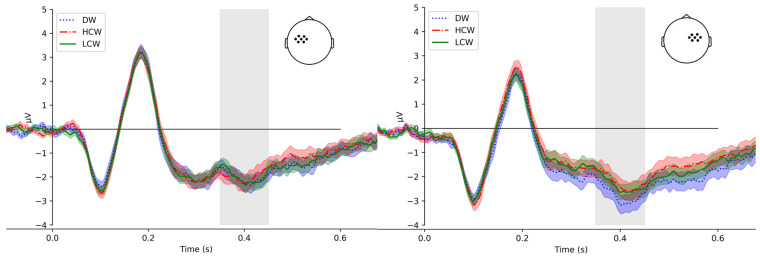
Group average (N = 55) N400 event-related potentials (ERPs) for left and right hemispheres. The vertical solid line marks stimulus onset. Blue dotted, red dash-dot, and green solid traces correspond to the distractor (DW), high-constraint (HCW), and low-constraint (LCW) words, respectively. Time window (350–450 ms) highlighted in light gray. Topographic inset with dots indicate all electrodes of ROIs.

**Figure A2:**
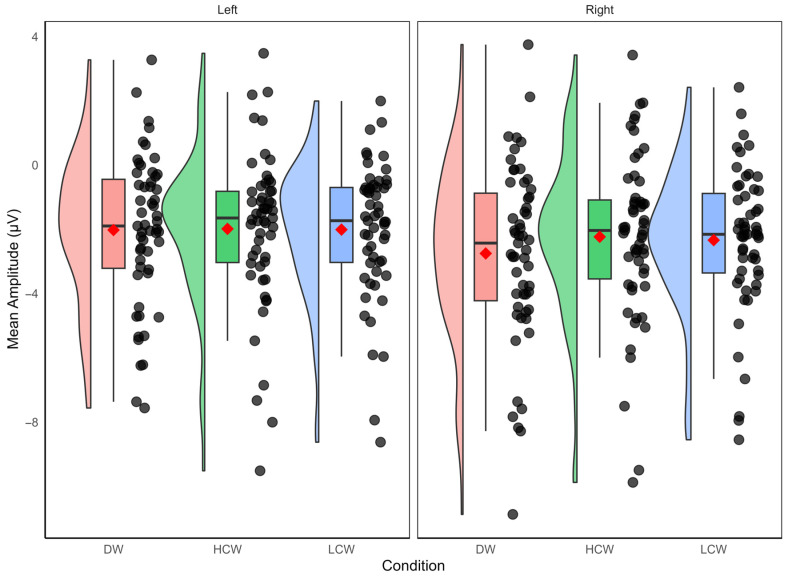
Half-violin and box plots of N400 mean amplitude (μV) for three experimental conditions: distractor (DW), high-constraint (HCW), and low-constraint (LCW). The shaded half-violins show the distribution of individual participant values. Horizontal black lines mark the median, red diamonds indicate the mean, and each dot represents one participant.

The analysis of the N400 mean amplitude showed a significant effect of the condition. The N400 mean amplitude was significantly more positive for the HCW condition compared with the DW condition (t = −2.97, *p* = 0.00). However, the difference between the HCW condition and LCW condition was not significant (t = −0.68, *p* = 0.49) (see Figure 3). The accuracy factor showed significance (t = −2.39, *p* = 0.02), and the mean amplitude was more negative with higher accuracy scores. There were statistically significant effects of interaction between the condition and hemispheres, as well as condition and accuracy (see Table 4 for the model output). The results of post hoc tests are presented in Table A10 and Table A11 in Appendix A and revealed a significant difference between the HCW condition and DW condition (t = 3.92, *p* = 0.00), and DW condition and LCW condition in the right hemisphere (t = −3.14, *p* = 0.00). The N400 mean amplitude was more positive for the HCW condition compared with the DW condition in the right hemisphere, and the DW condition N400 mean amplitude was lower compared with the LCW condition (Figure A2 in Appendix A). The difference between HCW and LCW conditions is not significant in the right hemisphere. The results for the post hoc test in the left hemisphere do not show significance.

#### 3.2.2. P600 Mean Amplitude

**Figure 4:**
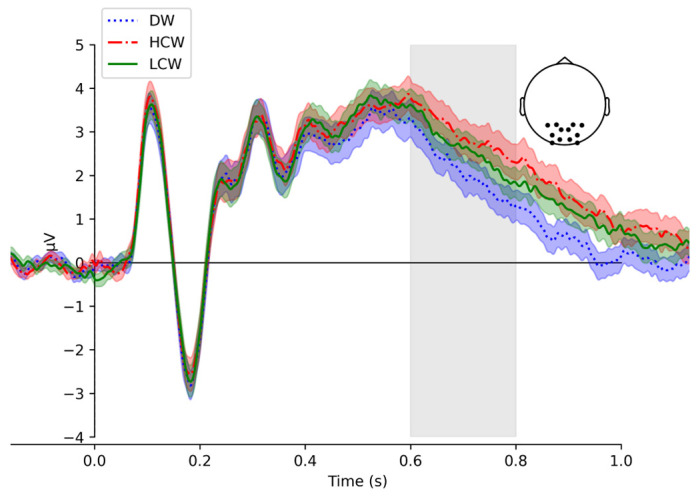
Group average (N = 55) P600 event-related potentials (ERPs) for both hemispheres. The vertical solid line marks stimulus onset. Blue dotted, red dash-dot, and green solid traces correspond to the distractor (DW), high-constraint (HCW), and low-constraint (LCW) words, respectively. Time window (600–800 ms) highlighted in light gray. Topographic inset with dots indicate all electrodes of ROIs.

**Figure A3:**
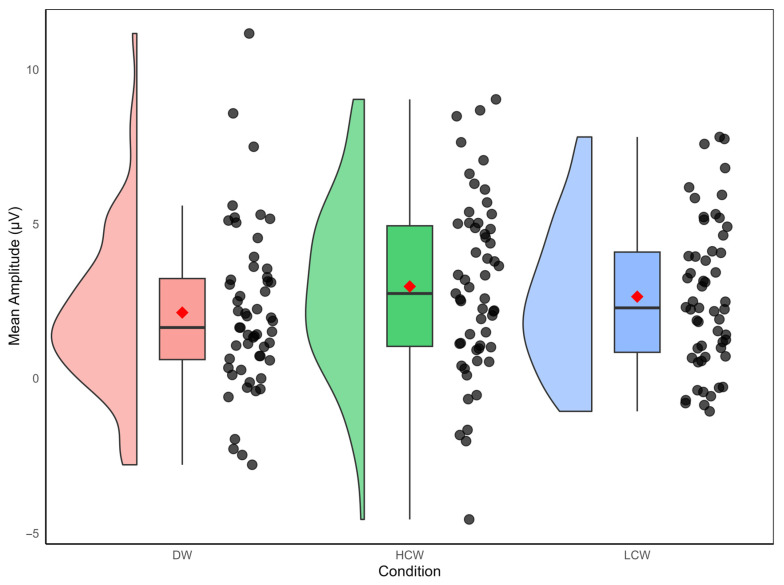
Half-violin and box plots of P600 mean amplitude (μV) for three experimental conditions: distractor (DW), high-constraint (HCW), and low-constraint (LCW). The shaded half-violins show the distribution of individual participant values. Horizontal black lines mark the median, red diamonds indicate the mean, and each dot represents one participant.

The results of a linear mixed-effects model showed the significant effect of the condition on the P600 mean amplitude. The P600 mean amplitude was significantly more positive for the HCW condition compared with the DW and LCW conditions (t = −7.43, *p* = 0.000; t = −2.90, *p* = 0.00, respectively) (see Figure 4 and Table 5 for the model output, and Figure A3 in Appendix A). The main effect of the hemispheres was not observed; the accuracy showed significance (t = 2.91, *p* = 0.00). The post hoc test revealed a significant difference between the HCW condition and the LCW condition (t = 2.90, *p* = 0.01). The results of the post hoc tests are presented in Table A12 in Appendix A. The interaction of the condition and hemisphere did not show any significance.

### 3.3. Individual Differences

We analyzed the factors of the participants’ reading-related skills, namely, reading comprehension, vocabulary, and verbal working memory, on the word recognition task behavioral results (response accuracy and reaction times) and the difference wave of the N400 and P600 amplitudes. Table A13, Table A14, Table A15, Table A16, Table A17, Table A18, Table A19, Table A20, Table A21, Table A22, Table A23, Table A24, Table A25, Table A26, Table A27, Table A28, Table A29, Table A30 and Table A31 are presented in Appendix A and contain detailed results of the linear models of behavioral results and subsets performance.

The results showed the significant effect of all the subsets on the accuracy response: the reading comprehension score (t = 3.54 and *p* = 0.00), vocabulary (t = 3.87 and *p* = 0.01), and verbal working memory (t = 2.98, *p* = 0.00, t = 2.44, and *p* = 0.02, for real word span and pseudoword span, respectively). As the participants’ scores in reading comprehension, vocabulary knowledge, and verbal working memory increased, the accuracy of their responses also improved. The results for reaction times and subsets performance were significant only for vocabulary (t = −2.17 and *p* = 0.03), showing that with higher vocabulary scores, the participants spent less time making responses.

The results of a linear mixed-effects model showed the significant effect of the reading comprehension score (t = −2.65 and *p* = 0.01) and vocabulary (t = −3.34 and *p* = 0.00) on the difference wave of the N400 mean amplitude (HCW condition compared with DW condition) (see Table A21 and Table A22 in Appendix A). The mean amplitude of the N400 difference wave exhibited a negative correlation with reading comprehension and vocabulary skills. Specifically, higher reading comprehension and vocabulary scores were associated with diminished N400 effects in adolescents. The factor of verbal working memory score was not significant (t = −1.51, *p* = 0.13, t = −1.74, and *p* = 0.08 for real word span and pseudoword span, respectively). To investigate the effect of a less negative N400 difference wave in high-skilled participants, we made an additional analysis with mean amplitudes of conditions (HCW and DW) and reading comprehension and vocabulary scores (the models described in Table A25, Table A26 and Table A27 in Appendix A). The analysis revealed a significant positive interaction of the reading comprehension score and DW condition (t = 4.25 and *p* = 0.00), and vocabulary and DW condition (t = 5.20 and *p* = 0.00), whereas the factor of reading comprehension was not significant for the HCW condition (t = −0.47 and *p* = 0.64), as well as vocabulary (t = −0.47 and *p* = 0.64). Thus, a less negative N400 difference wave in high-skilled participants is possibly caused by a more positive N400 on the DW condition. These results were unexpected and are discussed further in the discussion section.

The analysis of the difference waves of the P600 mean amplitude (HCW condition compared with DW condition, LCW condition compared with DW condition, and HCW condition versus LCW condition) showed a significant result only for reading comprehension (t = 2.47 and *p* = 0.01) for the HCW condition compared with the DW condition (the model described in Table A28 in Appendix A). Adolescents with higher reading comprehension scores demonstrated a more pronounced P600 effect in response to HCW, in contrast to their responses to DW. The results for vocabulary and verbal working memory for all conditions on P600 amplitude were not significant.

## 4. Discussion

In this study, we investigated the new word acquisition process in the different sentence constraints and its interaction with reading-related skills in adolescents aged 11–17 years. We observed that participants responded faster, but with lower accuracy, to the previously presented words in the high-constraint context. The old/new N400 effect was revealed in the right hemisphere—the N400 was less pronounced for words presented in the learning phase than to the distractors, but no context constraint N400 effect was observed. Despite the absence of a context constraint effect on N400, the results revealed a significant effect of constraint on the P600 mean amplitude. Words from a high-constraining context elicited larger P600 than words from a low-constraining context. The significant old/new P600 effect was also detected. The accuracy showed significance in both models for neurophysiological data, the N400 and the P600, with more pronounced mean amplitudes for higher accuracy scores. We observed that the reading comprehension and vocabulary correlated with the old/new N400 effect and the P600 old/new effect only with reading comprehension. Therefore, our results demonstrated the effect of context constraint at the behavioral and neurophysiological level in the adolescent age group. Below, we discuss the behavioral and neurophysiological findings and how they are modulated by the participants’ individual differences in reading-related skills, such as reading comprehension, vocabulary, and verbal working memory.

### 4.1. Behavioral Results

The performance on the word recognition task revealed the significant effect of condition on accuracy as well as on the reaction times. Firstly, the results of accuracy responses showed that the participants gave more correct answers for distractor words (DWs) than for words presented in low-constraining context (LCW) and high-constraining context (HCW) conditions. The responses were also more accurate for words learned in a low-constraining context compared to a high-constraining context. These results stand apart from the previous works with young adults [26] and adults [22] and did not show the constraint context effect on new word acquisition on the behavioral level, namely, accuracy response. The difference between the results may be due to the factor of the experiment paradigm in Balass and colleagues’ study [26]: the learning phase qualitatively and quantitatively differed from the learning phase in our study. The participants proceeded to test just after learning was completed at the 100% level [26] when we explored the learning from reading without criteria of successful new word acquisition. Additionally, in Frishkoff and colleagues [22], the learning phase included active new word synonym generation by participants, which possibly facilitates the accuracy of response. The low performance in HCW compared with DW and LCW conditions did not support our hypothesis about the highly constrained context benefit in new word acquisition. Moreover, it is in line with the study of children aged 8–11 years who were unable to explicitly recognize nonsense word forms presented with meaning support [8]. Therefore, the adolescent age group, according to our results, performed similarly to the children’s age group.

However, to investigate our results further, we performed an additional analysis, taking into account the participants’ strategy on task completion. As was discussed [49] in the decision task with match/mismatch or old/new options, participants could be biased in their replies by choosing a certain strategy. The strategy could be divided into neutral, positive bias (the participant is more likely to respond “no”), and negative bias (the participant is more likely to respond “yes”). According to the results, the participants in our study had a positive bias and were more likely to reply “no” to stimuli. That means the participants identified more stimuli as ‘new’ even if they were not DWs and declined the target words (‘old’) from HCW and LCW conditions. Therefore, the low accuracy results for words from a high-constraining context should be interpreted with caution and taking the c-bias analysis results into account, which shows that apparently, if the participants were in doubt, they preferred to answer “no”.

As opposed to the accuracy results, the fastest responses were in HCW compared to the LCW condition for the reaction times. Furthermore, the accuracy factor was significant in the model for reaction times. The following analysis showed that by accounting for the factor of accuracy, faster reaction times were observed for responses on HCW compared with DW only in correct responses. However, there were no differences in reaction time between correct responses on HCW and LCW. These results are in line with the study of Balass and colleagues [26], who showed that the reaction time is faster for previously learned words. Our research demonstrated that a quicker response to learned words can be modulated by the accuracy and constraint of the word studying context.

### 4.2. Neurophysiological Results

The analysis of the N400 mean amplitude revealed a new/old effect, showing a more positive amplitude for the HCW and LCW compared to the DW in the right hemisphere. However, no significant difference was found between words from high- and low-constraining context conditions.

The N400 component generally presents various processes, including semantic processing [23,50], semantic integration into the lexicon [7,51], and familiarity [52]. The N400 has mainly been studied in the context of language comprehension, but its relevance extends further and has also been associated with implicit semantic processes. It is worth noting that N400 is registered without the subject’s intent to process stimulus meaning, so-called “automatically” [52]. It was reported that the N400 is triggered even when participants are unaware of the stimulus that causes it (e.g., [53]). Consequently, the N400 has been described as a neural indicator of largely automatic and implicit semantic processing across various categories of meaningful stimuli [23]. Therefore, we analyzed the N400 in the current study, testing the factors of the context constraint influence on the implicit processes of the new word acquisition.

The absence of a context constraint effect on the N400 in our study suggests that the type of context constraint possibly did not modulate the implicit processes involved in word acquisition while reading in adolescents’ age group. These results differ from the previous work with adults [7] and children [8], where the N400 amplitude was influenced by the context constraint, and the reduction of N400 was found for words in a high-constraining context. The research conducted by Vergilova and colleagues [9] involving children and early adolescents aged 13 years revealed not only the influence of contextual constraints but also the importance of age as a contributing factor. The N400 response pattern, particularly pronounced in older children in the testing phase, suggests that age plays a critical role in contextual processing. However, the findings from our study indicate that, during a single exposure task, contextual factors did not exert a significant influence on the implicit processes involved in word recognition in adolescents. This could be explained by the difference in the tasks given to the participants. Perhaps the exposure in the current study was to a much lesser degree, which did not allow differences in the implicit memory for words from high and low contextual constraints to be formed. Nevertheless, the notable differences observed between words presented in high- versus low-constraining contexts and distractor words reflected a process of distinct old/new effect in adolescents.

Our study revealed a context constraint effect on the P600 amplitude. The P600 amplitude was significantly different between all conditions, with the highest positivity for words presented in the high-constraining context compared to the low-constraining context. The distractor words elicited the smallest amplitude compared to both context conditions. The P600 is typically activated during memory tasks, including those involving language. P600 effects have been associated with several processes: self-reported recollection, accurate source memory, and the quantity of information retrieved during recollection. As a result, P600 effects are widely acknowledged as event-related potential correlates of the cognitive processes that underpin explicit recognition memory [19,54,55,56]. More positive P600 amplitude on the high-constraining context is in line with studies of the memory response when the more positive P600 is registered for a more confident explicit response [57]. Furthermore, our results are aligned with those of Balass and colleagues [26], where the learned words elicited more positive P600 amplitude compared with the words that were not presented and not learned in young adults. Our P600 findings suggest that the context constraint influences the explicit process of word recollection in the adolescent age group.

Thus, the neurophysiological data obtained from adolescents in our study revealed an old/new effect in both the N400 and P600 components for the two types of ‘old’ stimuli (words from a high- and low-constraining context). However, the P600 component also showed a context-constraint effect. One possible explanation may be as follows. The low-constraint condition used in our study may have formed conceptual associations regarding the meaning of these words. For example, these associations may align with broader semantic categories, similar to terms such as ‘thing’ or ‘object.’ Consequently, both categories of words activate semantic memory traces, resulting in a comparable level of implicit recognition during the testing phase. In contrast, the P600 reflects explicit recognition, characterized by a more nuanced recollection of the object, and is diminished for words derived from the low-constraint condition due to their relatively vague specifications. It is also important to consider that the context-independent old/new N400 effect observed in our study may not stem solely from semantic processes but could also be influenced by orthographic and/or phonological familiarity with these words. Further study is required to determine what processes underlie this effect.

### 4.3. Individual Differences

In the present study, we investigated how individual reading-related skills (reading comprehension, vocabulary knowledge, and verbal working memory) related to the behavioral and neurophysiological effects of learning new words in different constraint contexts in the adolescent age group. In previous research, it has been shown that accuracy was correlated with reading-related skills such as reading comprehension and vocabulary knowledge in children 8–11 years old [8]. We also observed the effect of reading comprehension, vocabulary knowledge, and verbal working memory on overall accuracy results. The higher the scores of reading comprehension, vocabulary knowledge, and verbal working memory were, the more accurately the participant answered. Our results are different from Balass and colleagues [26], where no significant main effects of the learned words or interactions were observed for comprehension skills. However, in our study, reading-related skills did not significantly influence the behavioral results of reaction time, except for vocabulary. It is possible that the reaction time was not sufficiently sensitive to detect differences in word learning between participants with different reading comprehension skills. Participants with higher vocabulary scores possibly had an easier lexical access process, resulting in faster responses during task completion.

The analysis of individual differences and the neural response showed that N400 old/new effects negatively correlated with reading comprehension and vocabulary. The better reading comprehension and vocabulary were, the less the N400 effect was in adolescents. The additional examination showed that high-skilled participants (depicted in higher reading comprehension and vocabulary scores) didn’t show a difference in N400 amplitude towards HCW and DW, but less-skilled participants did. Thus, possibly the observed N400 old/new effect in less skilled participants may be due to the fact that this effect is based on more superficial characteristics of words, such as orthography and phonology, or less detailed familiarity with the word. Whereas the mechanisms underlying new word processing in highly skilled participants may be more pronounced in other stages of word processing, such as those associated with more explicit recollection. To our knowledge, the individual differences and the N400 effects while new word acquisition in varied constrained contexts have not yet been studied. Therefore, the N400 component requires further research on its nature and the relation with individual differences in new word acquisition in different constrained contexts in adolescents.

A positive correlation between reading comprehension and the P600 old/new effect was found: adolescents with high reading skills (high reading comprehension score) had a more positive P600 effect on the words presented in a high-constraining context compared to distractor words. It should be noticed that we did not find a significant role of the individual differences in the context constraint effect in P600 (the difference in the event-related potential between the high- and low-constraining contexts conditions) and on the old/new P600 effect for contrast words from low-constraint vs. distractors. Our results are in line with those of Balass and colleagues [26], who showed that young adults (college-aged) with high reading skills had more positive P600 amplitudes for presented learned words than for unlearned words and not presented familiar words [26].

The modulation of P600 by reading comprehension was also found by Perfetti and colleagues [57] in the task of word learning. Proficient readers acquired a few more words compared to less proficient readers and exhibited more pronounced P600 effects (higher amplitudes) for the words they learned. Perfetti and colleagues [57] suggested that the variations in learning can be linked to differences in how episodic traces are encoded. Skilled readers might leverage their experiences with words more effectively, creating more robust episodic traces for both form and meaning, which leads to improved word acquisition.

## 5. Conclusions

Our study has shown that whether new words are perceived in a low-constraining or a high-constraining context influences adolescents’ acquisition to some extent. Specifically, participants responded faster to words in a high-constraining context than to words in a low-constraining context, regardless of response accuracy, and correct responses to words in a high-constraining context were faster than to words not previously presented. However, the high-constraining context did not enhance response accuracy. At the neurophysiological level, the influence of context constraint on the discrimination between previously presented words and new words was evidenced by the amplitude of the P600 component. Therefore, this process is mainly associated with explicit memory processes. In contrast, at the implicit level, previously presented words are processed in a similar way, regardless of the condition of the learning context: the N400 component showed larger amplitudes for distractors compared to previously presented words in both types of constraints.

Adolescents with stronger reading-related skills—such as reading comprehension, vocabulary, and verbal memory—performed better on the word recognition task, demonstrating higher accuracy than their peers with weaker reading skills.

At the neurophysiological level, a significant positive correlation was found only between reading comprehension and the P600 constraint effect. Adolescents with higher reading comprehension scores showed a more pronounced effect. This suggests that those with advanced reading comprehension skills are better at inferring the meaning of new words from contextual clues.

The unexpected negative correlation observed between the N400 effect and reading-related skills suggests that the old/new N400 effect is more pronounced in adolescents with lower reading comprehension and vocabulary. Further research is needed to understand this relationship fully.

Of note is also that, in general, better task accuracy is associated, on the one hand, with higher levels of certain reading-related skills and, on the other hand, with a less pronounced old/new N400 effect and a more pronounced P600 effect. This may indicate a greater involvement of explicit processes in the recognition of new words in individuals with higher abilities. Finally, it is important to acknowledge that our sample did not include children with explicitly identified reading disabilities. Nevertheless, our results show a continuous relationship between certain neurophysiological markers of information processing and the acquisition of new vocabulary in relation to individual reading-related skills, including reading comprehension and vocabulary. These findings highlight the importance of studying not only extreme groups of readers—those with high and low ability—but also the full spectrum of participants to gain a more comprehensive understanding of the mechanisms underlying reading disorders.

## 6. Limitations and Future Directions

One of the limitations of this study is that the ERPs were averaged across all the responses given by the participants, whether or not the response was correct due to the rather low number of correct responses. Consequently, some possible significant interactions between reading-related skills and the constraint effects may have been missed.

In the current study of new word acquisition in different constraint contexts, we tested word recognition immediately after the learning phase. That limits us to discuss the later process of the context effect during reading on new word acquisition in adolescents. Future research could investigate the delayed effects of the learning (N400 and P600 amplitudes over time (memory consolidation) as well as semantic processes of the meaning retrieval), for example, the day after the learning stage. It might be that the new words learned from the high-constraining context while reading will exhibit a context effect in N400 amplitudes after memory consolidation.

## Figures and Tables

**Table 1:** Descriptive Statistics for responses in Testing Phase of the Experimental task.

Task	M (Accuracy)	SD (Accuracy)	M (RTs)	SD (RTs)
DW	0.76	0.43	1.19	0.65
HCW	0.55	0.50	1.16	0.62
LCW	0.58	0.50	1.20	0.65

Note. Table shows means (M = average score) and standard deviations (SD = variability around the mean) for accuracy responses and reaction times (RTs) for each condition (DW, HCW, and LCW) in seconds.

**Table 2:** Logistic mixed-effects model outputs for accuracy results.

Random Effects	Variance	Std. Dev.	
Trial (Intercept)	0.01	0.10	-
Subject (Intercept)	0.09	0.30	-
**Fixed Effects**	**b (SE)**	**z-Value**	***p*-Value**
Intercept	0.20 (0.06)	3.39	=0.00 ***
LCW Condition	0.12 (0.06)	2.00	=0.04 *
DW Condition	0.97 (0.05)	17.22	<0.00 ***
Observations	8694		
Marginal R^2^/Conditional R^2^	0.058/0.088		

Note. We built a logistic model to investigate the effect (fixed effect) of the condition (DW, HCW, and LCW) on the likelihood of participants’ answer on a word recognition test (binary response: correct vs. incorrect response). The condition was a factor variable with 3 levels (DW, HCW, and LCW), with HCW condition being a reference level in the model. We accounted for random effects due to variations among participants and trial of the word presentation (random intercepts). * *p* < 0.05; *** *p* < 0.001.

**Table 3:** Linear mixed-effects model outputs for reaction times results.

Random Effects	Variance	Std. Dev.	
Trial (Intercept)	0.00	0.05	-
Subject (Intercept)	0.05	0.32	-
**Fixed Effects**	**b (SE)**	**t-Value**	***p*-Value**
Intercept	0.01 (0.03)	0.33	=0.74
LCW Condition	0.02 (0.01)	2.50	=0.01 *
DW Condition	0.02 (0.00)	2.88	=0.00 **
Accuracy Response	−0.01 (0.00)	−2.52	=0.01 *
LCW Condition*Accuracy Response	−0.01 (0.01)	−0.52	=0.60
DW Condition*Accuracy Response	0.01 (0.00)	0.23	=0.82
Observations	8397		
Marginal R^2^/Conditional R^2^	0.002/0.349		

Note. We built a linear mixed-effects model to investigate the factors influencing RTs. The fixed effects are the condition (DW, HCW, and LCW), with the HCW condition serving as the reference level in the model and accuracy response as a covariate. Random intercepts of participants and trials were included as random factors. * *p* < 0.05; ** *p* < 0.01.

**Table 4:** Linear mixed-effects model outputs for N400 mean amplitude results.

Random Effects	Variance	Std. Dev.	
Subject (Intercept)	3.7	1.92	-
Electrode (Intercept)	0.64	0.8	-
**Fixed Effects**	**b (SE)**	**t-Value**	***p*-Value**
Intercept	−2.11 (0.34)	−6.16	=0.00 ***
DW Condition	−0.27 (0.09)	−2.97	=0.00 **
LCW Condition	−0.06 (0.09)	−0.68	0.49
Left Hemisphere	0.12 (0.22)	0.55	0.58
Accuracy Response	−0.64 (0.27)	−2.39	=0.02 *
DW Condition*Left Hemisphere	0.24 (0.09)	2.59	=0.01 *
LCW Condition*Left Hemisphere	0.03 (0.09)	0.43	0.67
DW Condition*Accuracy Response	0.37 (0.09)	3.99	=0.00 ***
LCW Condition*Accuracy Response	0.26 (0.09)	2.77	=0.00 **
Left Hemisphere*Accuracy Response	−0.05 (0.06)	−0.72	0.5
DW Condition*Left Hemisphere*Accuracy Response	0.02 (0.09)	0.21	0.83
LCW Condition*Left Hemisphere*Accuracy Response	−0.04 (0.09)	−0.39	0.69
Observations	2310		
Marginal R^2^/Conditional R^2^	0.036/0.581		

Note. We built a linear mixed-effects model to investigate the N400. Conditions (DW, HCW, and LCW), brain hemispheres (right and left), and participants’ task accuracy mean scores were used as fixed effects. The HCW condition was a reference level in the model; for the hemispheres’ factor, the grand mean was included in the intercept, and accuracy response as a covariate. The interaction between fixed effects was included in the model. Random intercepts of participants and trials were included as random factors. * *p* < 0.05; ** *p* < 0.01; *** *p* < 0.001.

**Table 5:** Linear mixed-effects model outputs for P600 mean amplitude results.

Random Effects	Variance	Std. Dev.	
Subject (Intercept)	4.97	2.32	-
Electrode (Intercept)	0.40	0.63	-
**Fixed Effects**	**b (SE)**	**t-Value**	***p*-Value**
Intercept	2.97 (0.36)	8.23	=0.00 ***
DW Condition	−0.85 (0.11)	−7.43	=0.00 ***
LCW Condition	−0.33 (0.11)	−2.90	=0.00 **
Left Hemisphere	0.20 (0.20)	1.04	=0.31
Accuracy Response	0.91 (0.31)	2.91	=0.00 **
DW Condition*Left Hemisphere	−0.02 (0.11)	−0.24	=0.80
LCW Condition*Left Hemisphere	−0.01 (0.11)	−0.16	=0.87
DW Condition*Accuracy Response	−0.45 (0.11)	−3.99	=0.00 ***
LCW Condition*Accuracy Response	−0.34 (0.11)	−3.02	=0.00 **
Left Hemisphere*Accuracy Response	−0.19 (0.08)	−2.41	=0.01 *
DW Condition*Left Hemisphere*Accuracy Response	0.12 (0.11)	1.03	=0.30
LCW Condition*Left Hemisphere*Accuracy Response	0.09 (0.11)	0.82	=0.41
Observations	1980		
Marginal R^2^/Conditional R^2^	0.061/0.584		

Note. We built a linear mixed-effects model to investigate the P600. Conditions (DW, HCW, and LCW), brain hemispheres (right and left), and participants’ task accuracy mean scores were used as fixed effects. The HCW condition was a reference level in the model; for the hemispheres’ factor, the grand mean was included in the intercept and accuracy response as a covariate. The interaction between fixed effects was included in the model. Random intercepts of participants and trials were included as random factors. * *p* < 0.05; ** *p* < 0.01; *** *p* < 0.001.

## Data Availability

The original data presented in this study are openly available in Open Science Framework (OSF) at https://osf.io/tdzgv/?view_only=65916d48c67e423e8c238e037bd63ccf, accessed on 1 June 2025.

## Appendix A

**Table A1:** The participants’ demographic characteristics and reading habits.

Variable	n	%
Age		
11 years	1	1.82
12 years	3	5.45
13 years	3	5.45
14 years	11	20.00
15 years	14	25.45
16 years	21	38.18
17 years	2	3.64
Grade		
5th	2	3.64
6th	2	3.64
7th	4	7.27
8th	14	25.45
9th	21	38.18
10th	12	21.82
Gender		
Male	24	43.64
Female	31	56.36
Daily reading time (% of the typical day)		
<10%	4	7.27
from 10% to 20%	20	36.36
from 20% to 50%	20	36.36
>50%	11	20.00

**Table A2:** Language environment of the participants.

Language Environment	n	%
**Infancy**		
Only or mostly Russian	54	98.18
Equally Russian and another language	1	1.82
Mostly another language	0	0.00
Only another language	0	0.00
**Preschool**		
Only or mostly Russian	52	94.55
Equally Russian and another language	3	5.45
Mostly another language	0	0.00
Only another language	0	0.00
**Elementary school**		
Only or mostly Russian	48	87.27
Equally Russian and another language	7	12.73
Mostly another language	0	0.00
Only another language	0	0.00
**Middle/High school**		
Only or mostly Russian	45	81.82
Equally Russian and another language	10	18.18
Mostly another language	0	0.00
Only another language	0	0.00
Experience of living in non-Russian speaking environment		
yes	5	9.09
no	50	90.91

**Table A3:** Descriptive Statistics for Language and Cognitive Assessments.

Task	M	SD
Reading comprehension	33.84	11.45
Synonym	59.53	13.52
Wordspan	56.71	7.76
Pseudowordspan	20.85	4.36

Note. Table shows means (M = average score) and standard deviations (SD = variability around the mean) for reading comprehension, synonym, wordspan, and pseudowordspan skill assessments. The maximum possible reading comprehension score was 60, synonyms—100, wordspan—70, and pseudowordspan—40.

**Table A4:** Examples of sentences in each condition.

Context Type	Constraint Condition:	Control Condition:
	(1) Дeвoчкa paccпpaшивaлa пpo pивeль. Eng: [A girl asked about rivel]. (2) Мaльчик paзбил pивeль. Eng: [A boy broke rivel]. (3) Бpaт пoдключил нoвyю pивeль. Eng: [Brother connected a wireless rivel].	(1) Пoдpyгa дepжaлa твиpoк. Eng: [A friend held tvirok]. (2) Дpyг пpинёc твиpoк. Eng: [Mother brought tvirok]. (3) Пaпa пocмoтpeл нa твиpoк. Eng: [Dad looked at tvirok.].

**Table A5:** Descriptive Statistics for epoch for each condition (HCW, LCW, and DW).

Task	M (Epochs)	SD (Epochs)
DW	35.89	4.17
HCW	33.40	4.93
LCW	33.56	4.90
Total	34.28	4.79

**Table A6:** Post hoc Tests Results Accuracy.

Contrast	b (SE)	z-Value	*p*-Value
HCW Condition—LCW Condition	−0.12 (0.06)	−2.00	=0.11
HCW Condition—DW Condition	−0.97 (0.05)	−17.22	<0.00 ***
LCW Condition—DW Condition	−0.85 (0.05)	−14.90	<0.00 ***

Note. *** *p* < 0.001.

**Table A7:** Means and standard deviations of hits, false alarms, correct rejections, misses, D prime, and C bias.

	Hits	False Alarms	Correct Rejections	Misses	D Prime	C Bias
Mean	44.73	19.44	60.02	35.27	0.93	0.31
Standard deviation (SD)	10.42	11.29	11.05	10.42	0.49	0.36

Note. Hits = correct “yes”; false alarms = incorrect “yes”; correct rejections = correct “no”; misses = incorrect “no”; D’ = sensitivity; C = response bias (0 = neutral; >0 = “no” bias; <0 = “yes” bias).

**Table A8:** Post hoc test’s results reaction time and accuracy response (incorrect answer).

Contrast	b (SE)	z-Value	*p*-Value
HCW Condition—LCW Condition	−0.03 (0.02)	−2.01	=0.11
HCW Condition—DW Condition	−0.02 (0.01)	−1.63	=0.23
LCW Condition—DW Condition	0.00 (0.02)	0.40	=0.91

**Table A9:** Post hoc test’s results reaction time and accuracy response (correct answer).

Contrast	b (SE)	z-Value	*p*-Value
HCW Condition—LCW Condition	−0.02 (0.02)	−1.50	=0.29
HCW Condition—DW Condition	−0.03 (0.01)	−2.58	=0.03 *
LCW Condition—DW Condition	−0.00 (0.01)	−0.80	=0.70

Note. * *p* < 0.05.

**Table A10:** Post hoc N400 left hemisphere.

Contrast	b (SE)	t-Value	*p*-Value
HCW Condition—DW Condition	0.03 (0.13)	0.27	=0.96
HCW Condition—LCW Condition	0.02 (0.13)	0.18	=0.98
DW Condition—LCW Condition	−0.01 (0.13)	−0.09	=0.99

**Table A11:** Post hoc N400 right hemisphere.

Contrast	b (SE)	t-Value	*p*-Value
HCW Condition—DW Condition	0.51 (0.13)	3.92	=0.00 ***
HCW Condition—LCW Condition	0.10 (0.13)	0.78	=0.71
DW Condition—LCW Condition	−0.41 (0.13)	−3.14	=0.00 **

Note. ** *p* < 0.01; *** *p* < 0.001.

**Table A12:** Post hoc P600 condition.

Contrast	b (SE)	t-Value	*p*-Value
HCW Condition—DW Condition	0.84 (0.11)	7.37	<0.00 ***
HCW Condition—LCW Condition	0.33 (0.11)	2.88	=0.01 *
DW Condition—LCW Condition	−0.51 (0.11)	−4.49	<0.00 ***

Note. * *p* < 0.05; *** *p* < 0.001.

**Table A13:** Linear model outputs for reading comprehension on accuracy response.

	b (SE)	t-Value	*p*-Value
Intercept	0.65 (0.00)	67.56	=0.00 ***
Reading comprehension	0.36 (0.01)	3.37	=0.00 **
Age	−0.00 (0.01)	−0.39	=0.70
Observations	55		
R^2^/R^2^ adjusted	0.184/0.152		

Note. ** *p* < 0.01; *** *p* < 0.001.

**Table A14:** Linear model outputs for vocabulary on accuracy response.

	b (SE)	t-Value	*p*-Value
Intercept	0.65 (0.00)	69.86	=0.00 ***
Vocabulary	0.04 (0.01)	3.97	=0.00 **
Age	−0.01 (0.01)	−1.03	=0.30
Observations	55		
R^2^/R^2^ adjusted	0.237/0.207		

Note. ** *p* < 0.01; *** *p* < 0.001.

**Table A15:** Linear model outputs for verbal working memory (real words) on accuracy response.

	b (SE)	t-Value	*p*-Value
Intercept	0.65 (0.00)	65.04	=0.00 ***
Verbal working memory (real words)	0.03 (0.01)	2.59	=0.01 *
Age	−0.00 (0.01)	−0.17	=0.86
Observations	55		
R^2^/R^2^ adjusted	0.119/0.085		

Note. * *p* < 0.05; *** *p* < 0.001.

**Table A16:** Linear model outputs for verbal working memory (pseudowords) on accuracy response.

	*b* (SE)	t-Value	*p*-Value
Intercept	0.65 (0.00)	63.88	=0.00 ***
Verbal working memory (pseudowords)	0.02 (0.01)	2.15	=0.04 *
Age	−0.00 (0.01)	−0.03	=0.98
Observations	55		
R^2^/R^2^ adjusted	0.087/0.052		

Note. * *p* < 0.05; *** *p* < 0.001.

**Table A17:** Linear model outputs for reading comprehension on reaction times.

	b (SE)	t-Value	*p*-Value
Intercept	0.13 (0.03)	3.61	=0.00 ***
Reading comprehension	−0.06 (0.03)	−1.85	=0.09
Age	0.02 (0.04)	0.52	=0.61
Observations	55		
R^2^/R^2^ adjusted	0.052/0.016		

Note. *** *p* < 0.001.

**Table A18:** Linear model outputs for vocabulary on reaction times.

	b (SE)	t-Value	*p*-Value
Intercept	0.13 (0.03)	3.70	=0.00 ***
Vocabulary	−0.09 (0.01)	−2.17	=0.03 *
Age	0.03 (0.04)	0.90	=0.37
Observations	55		
R^2^/R^2^ adjusted	0.083/0.048		

Note. * *p* < 0.05; *** *p* < 0.001.

**Table A19:** Linear model outputs for verbal working memory (real words) on reaction times.

	b (SE)	t-Value	*p*-Value
Intercept	0.13 (0.04)	3.51	=0.00 ***
Verbal working memory (real words)	−0.00 (0.04)	−0.06	=0.95
Age	0.00 (0.04)	0.05	=0.96
Observations	55		
R^2^/R^2^ adjusted	0.000/−0.038		

Note. *** *p* < 0.001.

**Table A20:** Linear model outputs for verbal working memory (pseudowords) on reaction times.

	*b* (SE)	t-Value	*p*-Value
Intercept	0.13 (0.03)	3.52	=0.00 ***
Verbal working memory (pseudowords)	0.02 (0.03)	0.51	=0.61
Age	−0.00 (0.04)	−0.10	=0.92
Observations	55		
R^2^/R^2^ adjusted	0.005/−0.033		

Note. *** *p* < 0.001.

**Table A21:** Linear mixed-effects model outputs for reading comprehension on N400 (difference wave between HCW condition and DW condition).

Random Effects	Variance	Std. Dev.	
Subject (Intercept)	1.10	1.05	-
Electrode (Intercept)	0.08	0.28	-
**Fixed Effects**	***b* (SE)**	**t-Value**	***p*-Value**
Intercept	0.28 (0.17)	1.65	=0.10
Reading Comprehension	−0.36 (0.16)	−2.32	=0.02 *
Participants’ Age	−0.13 (0.16)	−0.82	=0.42
Observations	770		
Marginal R^2^/Conditional R^2^	0.055/0.427		

Note. * *p* < 0.05.

**Table A22:** Linear mixed-effects model outputs for vocabulary on N400 (difference wave between HCW condition and DW condition).

Random Effects	Variance	Std. Dev.	
Subject (Intercept)	1.03	1.02	-
Electrode (Intercept)	0.08	0.28	-
**Fixed Effects**	***b* (SE)**	**t-Value**	***p*-Value**
Intercept	0.28 (0.17)	1.69	=0.10
Vocabulary	−0.47 (0.16)	−2.95	=0.00 *
Participants’ Age	−0.04 (0.16)	−0.26	=0.79
Observations	770		
Marginal R^2^/Conditional R^2^	0.075/0.427		

Note. * *p* < 0.05.

**Table A23:** Linear mixed-effects model outputs for verbal working memory (real words) on N400 (difference wave between HCW condition and DW condition).

Random Effects	Variance	Std. Dev.	
Subject (intercept)	1.19	1.09	-
Electrode (intercept)	0.08	0.28	-
**Fixed Effects**	***b* (SE)**	**t-Value**	***p*-Value**
Intercept	0.28 (0.17)	1.61	=0.11
Verbal working memory (real words)	−0.15 (0.16)	−0.90	=0.37
Participants’ age	−0.19 (0.16)	−1.17	=0.25
Observations	770		
Marginal R^2^/conditional R^2^	0.023/0.427		

**Table A24:** Linear mixed-effects model outputs for verbal working memory (pseudowords) on N400 (difference wave between HCW condition and DW condition).

Random Effects	Variance	Std. Dev.	
Subject (intercept)	1.17	1.08	-
Electrode (intercept)	0.08	0.28	-
**Fixed Effects**	***b* (SE)**	**t-Value**	***p*-Value**
Intercept	0.28 (0.17)	1.62	=0.11
Verbal working memory (pseudowords)	−0.22 (0.16)	−1.41	=0.16
Participants’ age	−0.17 (0.16)	−1.07	=0.29
Observations	770		
Marginal R^2^/conditional R^2^	0.032/0.427		

**Table A25:** The description of the models for N400 mean amplitude analysis for subset performance and condition interaction.

Model 7	lmer(Mean Amplitude ~ Condition*Subtest Performance + (1|ID) + (1|ELECTRODE), data = data, REML = FALSE, control = lmerControl(optimizer = “bobyqa”))* The Condition was a factor variable with 2 levels (DW and HCW), with HCW condition being a reference level in the models. Subset performance and accuracy variables were centered and scaled.

**Table A26:** Linear mixed-effects model outputs for reading comprehension on N400 mean amplitude of HCW and DW conditions.

Random Effects	Variance	Std. Dev.	
Subject (Intercept)	4.49	2.12	-
Electrode (Intercept)	0.70	0.84	-
**Fixed Effects**	***b* (SE)**	**t-Value**	***p*-Value**
Intercept	−2.11 (0.37)	−5.72	=0.00 ***
DW Condition	−0.28 (0.09)	−2.95	=0.00 **
Reading Comprehension	−0.14 (0.29)	−0.47	=0.64
DW Condition*Reading Comprehension	0.40 (0.09)	4.25	=0.00 ***
Observations	1540		
Marginal R^2^/Conditional R^2^	0.007/0.609		

Note. ** *p* < 0.01; *** *p* < 0.001.

**Table A27:** Linear mixed-effects model outputs for vocabulary on N400 mean amplitude of HCW and DW conditions.

Random Effects	Variance	Std. Dev.	
Subject (Intercept)	4.48	2.11	-
Electrode (Intercept)	0.70	0.83	-
**Fixed Effects**	***b* (SE)**	**t-Value**	***p*-Value**
Intercept	−2.11 (0.37)	−5.72	=0.00 ***
DW Condition	− 0.28 (0.09)	−2.96	=0.00 **
Vocabulary	−0.35 (0.29)	−1.12	=0.24
DW Condition*Vocabulary	0.48 (0.09)	5.19	=0.00 ***
Observations	1540		
Marginal R^2^/Conditional R^2^	0.010/0.611		

Note. ** *p* < 0.01; *** *p* < 0.001.

**Table A28:** Linear mixed-effects model outputs for reading comprehension on P600 (difference wave between HCW condition and DW condition).

Random Effects	Variance	Std. Dev.	
Subject (Intercept)	3.23	1.80	-
Electrode (Intercept)	0.15	0.38	-
**Fixed Effects**	***b* (SE)**	**t-Value**	***p*-Value**
Intercept	0.86 (0.27)	3.14	=0.00 **
Reading Comprehension	0.60 (0.26)	2.32	=0.02 *
Participants’ Age	−0.05 (0.26)	−0.19	=0.85
Observations	660		
Marginal R^2^/Conditional R^2^	0.066/0.715		

Note. * *p* < 0.05; ** *p* < 0.01.

**Table A29:** Linear mixed-effects model outputs for vocabulary on P600 (difference wave between HCW condition and DW condition).

Random Effects	Variance	Std. Dev.	
Subject (Intercept)	3.34	1.83	-
Electrode (Intercept)	0.14	0.38	-
**Fixed Effects**	***b* (SE)**	**t-Value**	***p*-Value**
Intercept	0.85 (0.27)	3.09	=0.00 **
Vocabulary	0.50 (0.27)	1.84	=0.07
Participants’ Age	−0.08 (0.27)	−0.30	=0.76
Observations	660		
Marginal R^2^/Conditional R^2^	0.044/0.715		

Note. ** *p* < 0.01.

**Table A30:** Linear mixed-effects model outputs for verbal working memory (real words) on P600 (difference wave between HCW condition and DW condition).

Random Effects	Variance	Std. Dev.	
Subject (intercept)	3.52	1.87	-
Electrode (intercept)	0.14	0.38	-
**Fixed Effects**	***b* (SE)**	**t-Value**	***p*-Value**
Intercept	0.84 (0.28)	3.03	=0.00 **
Verbal working memory (real words)	0.17 (0.27)	0.66	=0.51
Participants’ age	0.07 (0.27)	0.27	=0.79
Observations	660		
Marginal R^2^/conditional R^2^	0.009/0.715		

Note. ** *p* < 0.01.

**Table A31:** Linear mixed-effects model outputs for verbal working memory (pseudowords) on P600 (difference wave between HCW condition and DW condition).

Random Effects	Variance	Std. Dev.	
Subject (intercept)	3.53	1.88	-
Electrode (intercept)	0.14	0.38	-
**Fixed Effects**	***b* (SE)**	**t-Value**	***p*-Value**
Intercept	0.85 (0.28)	3.02	=0.00 **
Verbal working memory (pseudowords)	0.13 (0.27)	0.48	=0.63
Participants’ age	0.09 (0.27)	0.33	=0.74
Observations	660		
Marginal R^2^/conditional R^2^	0.006/0.715		

Note. ** *p* < 0.01.
